# Attention LinkNet-152: a novel encoder-decoder based deep learning network for automated spine segmentation

**DOI:** 10.1038/s41598-025-95243-z

**Published:** 2025-04-16

**Authors:** Aqsa Dastgir, Wang Bin, Muhammad Usman Saeed, Jinfang Sheng, Luo Site, Haseeb Hassan

**Affiliations:** 1 School of Computer Science and Engineering, Central South University, Changsha, 410083 China; 2 College of Health Science and Environmental Engineering, Shenzhen Technology University, Shenzhen, 518118 China

**Keywords:** Neuroscience, Diseases, Health care, Health occupations, Medical research, Neurology, Mathematics and computing

## Abstract

Segmenting the spine from CT images is crucial for diagnosing and treating spine-related conditions but remains challenging due to the spine’s complex anatomy and imaging artifacts. This study introduces a novel encoder-decoder-based deep learning approach, named LinkNet-152, tailored for automated spine segmentation. The model integrates a modified EfficientNetB7 encoder with attention modules to enhance feature extraction by focusing on regions of interest. The decoder leverages a modified LinkNet architecture, replacing ResNet34 with the deeper ResNet152 to improve feature extraction and segmentation accuracy. Additionally, gradient sensitivity-based pruning is applied to optimize the model’s complexity and computational efficiency. Evaluated on the VerSe 2019 and VerSe 2020 datasets, the proposed model achieves superior performance, with a Dice coefficient of 96.85% and a Jaccard index of 95.37%, outperforming state-of-the-art methods. These results highlight the model’s effectiveness in addressing the challenges of spine segmentation and its potential to advance clinical applications.

## Introduction

The human spine is a critical structure that provides support and stability to the body while protecting the spinal cord. It is composed of a series of vertebrae, intervertebral discs, and ligaments, all working together to enable movement, flexibility, and load-bearing functions. The spine plays a vital role in maintaining posture, facilitating motion, and safeguarding neural pathways, making it one of the most crucial components of the musculoskeletal system. Due to its complex structure and essential functions, any abnormalities or injuries to the spine can lead to significant health issues, including pain, mobility restrictions, and neurological deficits.

Spine segmentation is a vital task in medical imaging, particularly for diagnosing and treating spinal disorders such as herniated discs, scoliosis, and spinal tumors^1^. Accurate segmentation of spinal structures from medical images, such as CT or MRI scans, allows clinicians to precisely identify and assess pathological conditions, plan surgeries, and monitor treatment outcomes^2^. It also aids in the development of computer-aided diagnosis (CAD) systems and the creation of patient-specific models for surgical simulations. The importance of spine segmentation extends beyond diagnostics, as it contributes to advancing research in spinal biomechanics, improving the understanding of spinal diseases, and enhancing the overall quality of patient care.

Despite its significance, spine segmentation presents numerous challenges due to the spine’s intricate anatomy, variability in shape and size among individuals, and the presence of artifacts and noise in medical images^3^. Traditional manual segmentation is time-consuming and prone to inter-observer variability, highlighting the need for automated and semi-automated methods. Recent advances in deep learning, particularly deep learning^4^, have shown great promise in overcoming these challenges by leveraging large datasets and powerful computational techniques to achieve high accuracy in spine segmentation. As the field progresses, the development of robust and efficient segmentation algorithms continues to be a key focus, aiming to bridge the gap between clinical needs and technological capabilities.

Defining the precise boundaries of individual vertebrae poses a challenge due to its intricate articulation, leading to instances of overlapping vertebrae during segmentation^5^. Although there is increasing attention on spine segmentation and vertebrae identification, the development of dependable and accurate spine segmentation methods remains elusive. Many techniques designed to segment spine patients with osteoporosis fractures encounter difficulties, as these patients often exhibit vertebral fractures at different stages and display spinal irregularities^6,7^. Given that the distinctive shapes can significantly deviate from.

the average shape, segmentation methods reliant on predetermined models may prove ineffective.

In this research, an automated deep-learning model for spine segmentation is proposed. It works in two phases; first, the EfficientNetB7 with attention module is used to extract features from CT images. The attention module helps to focus on the region of interest to extract prominent features. Secondly, the LinkNet-152 is used to predict the segmented spine. The standard architecture of LinkNet contains resnet34 as the encoder. However, resnet-152 is used in this research as an encoder in the LinkNet which helps in the final segmentation process. The key contributions of the proposed LinkNet-152 are:

- To proposed a deep learning model that helps for the segmentation of spine efficiently and accurately.
- To extract deep features from axial, coronal, and sagittal views, EfficientNetB7 is used by adding attention modules to focus on important features.
- Features from sagittal, axial, and coronal views are concatenated to form a 3D feature map, and given to the proposed LinkNet-152 for final segmentation.
- To get local and global features, ResNet-152 is used in the encoder part of the proposed LinkNet-152.

### Related work

Artificial intelligence algorithms leverage the power to accurately assess medical images like X-rays, MRIs, and CT scans. This proficiency contributes to the early identification and diagnosis of diseases, encompassing cancer and neurological conditions, facilitating prompt intervention. AI’s capability extends to scrutinizing patient data—comprising medical history, genetic details, and lifestyle elements—to craft tailored treatment strategies. This empowers healthcare practitioners to enhance treatment choices and elevate patient results.

Kim et al.^8^ proposed a CNN model for the segmentation of the spine. A U-net architecture was used to train the hierarchical data format. After training, data segmentation was applied to data by labeling it. A total of 344 CT images were used for the experiments. The results obtained by using this model was 90.4% dice score. A fully automated grading system was proposed by Tang et al.^9^ to deal with the three major problems related to spine stenosis. The study includes convolution neural networks for the automated vertebral segmentation and spinal stenosis grading. A high degree of performance of stenosis grading classes for both central spinal and foraminal locations was achieved.

In the realm of automated medicine, three-dimensional imagery has gained widespread application. Buerger et al.^10^ introduced a 4-stage methodology for segmenting the spine automatically. Initially, a U-Net was employed to create a coarse segmentation of the spine. The second step involved sampling image patches along this coarse segmentation, utilizing a secondary multi-class U-Net to generate a refined segmentation that encompassed individual labeling of crucial vertebrae and vertebral body landmarks. Step 3 focused on identifying and labeling landmark positions based on the classes derived in Step 2. Finally, Step 4 utilized the landmarks identified in Step 3 to initialize the MBS (model-based segmentation) vertebrae models and adapt these models to the combined vertebrae probability map from Step 2. This approach yielded a 90% success rate.

Syed et al.^4^ introduced a deep learning model based on patches, leveraging a stacked sparse auto-encoder to extract features from unlabeled data. This model divided 2D slices from a CT image into patches to extract relevant features, employing a sigmoid layer for the final vertebrae classification. Evaluation on three publicly available datasets demonstrated significantly improved dice score, achieving 90.2%. In a separate study, Zhang et al.^11^ proposed a U-shaped network algorithm for automating spine segmentation. To enhance the model’s performance while reducing computational cost, batch normalization was utilized. They also used MRI images to augment the dataset’s quality. Findings indicated that the proposed model surpassed U-net and FCN in both computational cost and accuracy.

For segmentation and spine parameter inspection a multitask neural network was proposed by Van et al.^12^ The MRNet was divided into two branches; one for the segmentation of lumbar vertebrae that gives the output to the second branch where classification and detection is done by supervised learning. The proposed model performed well even with the limited amount of data. Mustaq et al.^13^ used the YOLOv5 and HED U-net for the localization and segmentation of spine lumber. The lumbar vertebrae were localized by YOLOv5 and then HED U-Net, which is a combination of edge-base detection methods, was used for the segmentation. The proposed model obtained a mean average precision of 0.975.

## Methodology

Deep learning has revolutionized the field of medical image analysis^14^, including spine disease diagnosis^15,16^, brain tumor^17,18^, Alzheimer’s disease^19,20^, anemia detection^21^. The capacity to learn complicated patterns and characteristics over a large dataset has enhanced the precision, speed, and consistency of spine disease diagnosis. Deep learning algorithms can accurately segment different structures in spine images, such as vertebrae, intervertebral discs, and spinal canals. Automated segmentation reduces.

**Fig. 1:**
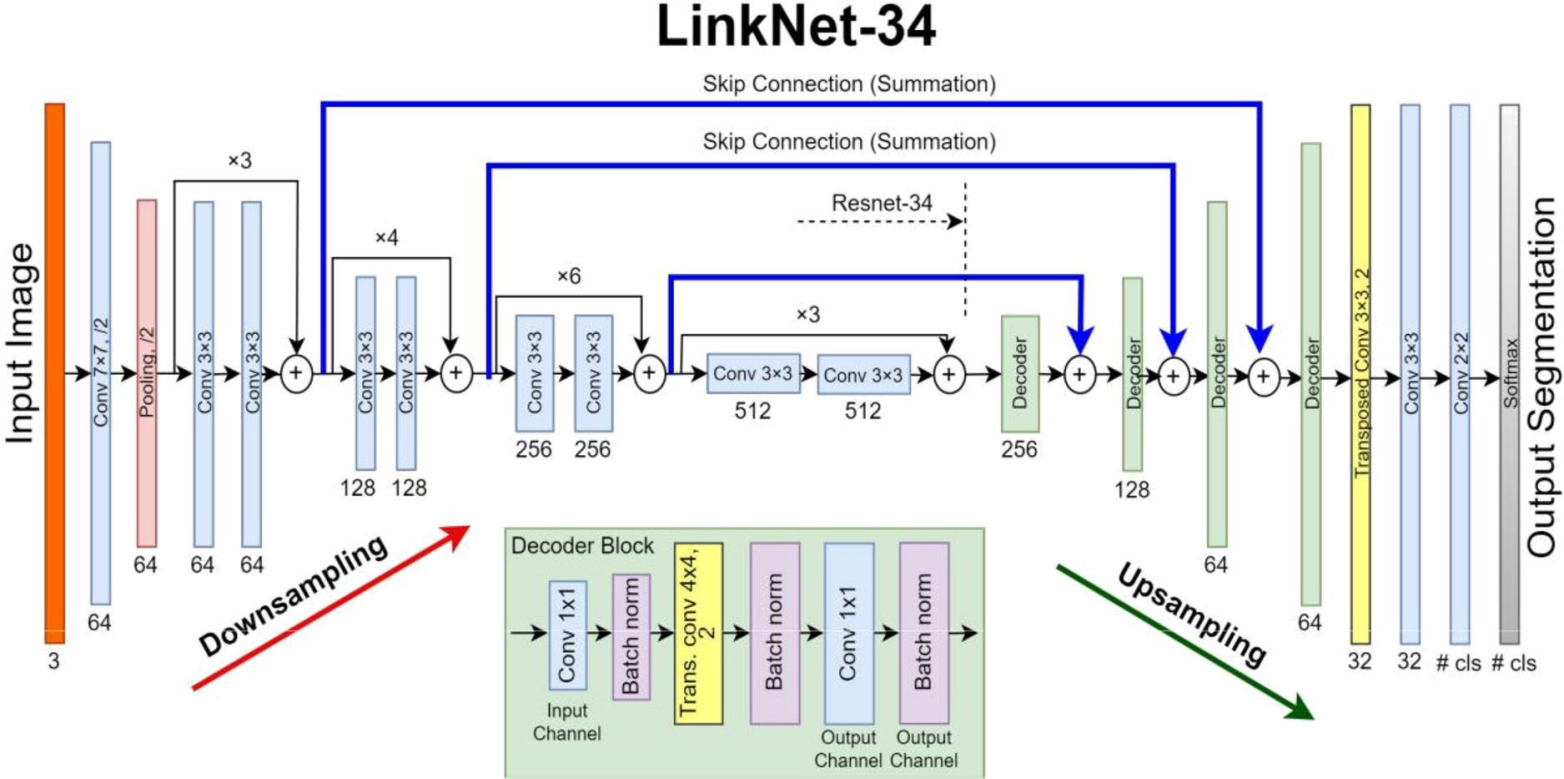
LinkNet-34 Model Architecture^22^.

the need for manual annotations, saving time and minimizing inter-observer variability, which is especially crucial in large-scale studies.

### LinkNet-34

LinkNet-34^22^ is a CNN architecture designed for semantic segmentation tasks in computer vision. It is an extension of the original LinkNet, which aimed to provide a simple and efficient architecture for accurate pixel-level image segmentation. LinkNet-34 is specifically built to be deeper and more powerful than its predecessor while maintaining the same design principles. The encoder segment of LinkNet-34 comprises a sequence of convolutional layers succeeded by batch normalization and ReLU activation functions. As it proceeds, it systematically diminishes the input’s spatial dimensions while amplifying the channel count. This layered structure allows the network to grasp intricate representations, encompassing both low-level and high-level features from the input image. The decoder part of LinkNet-34 is responsible for upsampling the feature maps from the encoder and generating the final segmentation map. It uses transposed convolution (also known as fractionally strided convolution or deconvolution) layers to increase the spatial resolution. Skip connections connect the decoder layers to corresponding encoder layers, allowing the decoder to incorporate fine-grained details from earlier stages of the network. Figure 1 shows the LinkNet-34 model.

### Feature Ppyramid network (FPN)

**Fig. 2:**
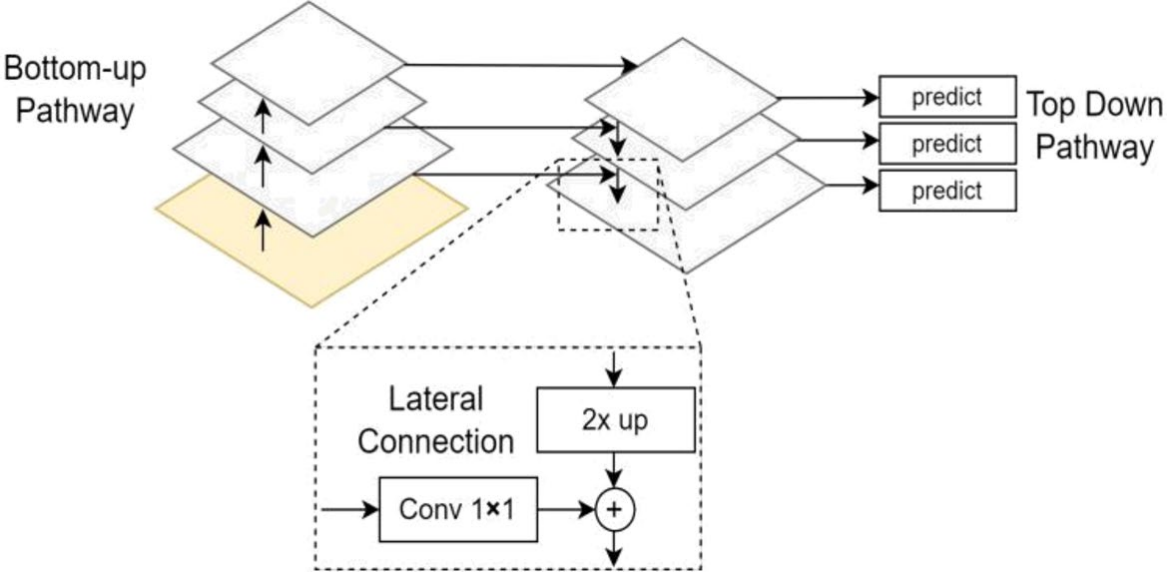
FPN Model Architecture^23^.

A FPN^23^ is a neural network architecture commonly used in computer vision tasks. It addresses the challenge of detecting objects at different scales within an image. FPNs are designed to capture and leverage multi-scale features from a single input image efficiently. The core idea behind FPN is to build a pyramid of feature maps with different spatial resolutions. Each level of the pyramid corresponds to a different scale of the input image. These feature maps capture information at various scales, from fine details to coarse structures. In semantic segmentation, FPN can be used to obtain multi-scale feature maps that help in accurately segmenting objects or regions of interest within an image. The fine-grained features from higher-resolution levels help preserve details, while the lower-resolution features provide context. The FPN model is shown in Fig. 2.

### PSPNet model

PSPNet, or Pyramid Scene Parsing Network^24^, is a deep learning model designed for the task of semantic scene parsing or scene segmentation. It was introduced to address the challenge of segmenting images into different object categories and regions, providing pixel-level labels for each part of the image. PSPNet was particularly influential in the field of computer vision and has been used in various applications, including autonomous driving, robotics, and image understanding. PSPNet follows the encoder-decoder architecture, similar to other semantic segmentation models. The encoder is responsible for extracting features from the input image, while the decoder upsamples the features to produce the final segmentation map. The core innovation of.

**Fig. 3:**
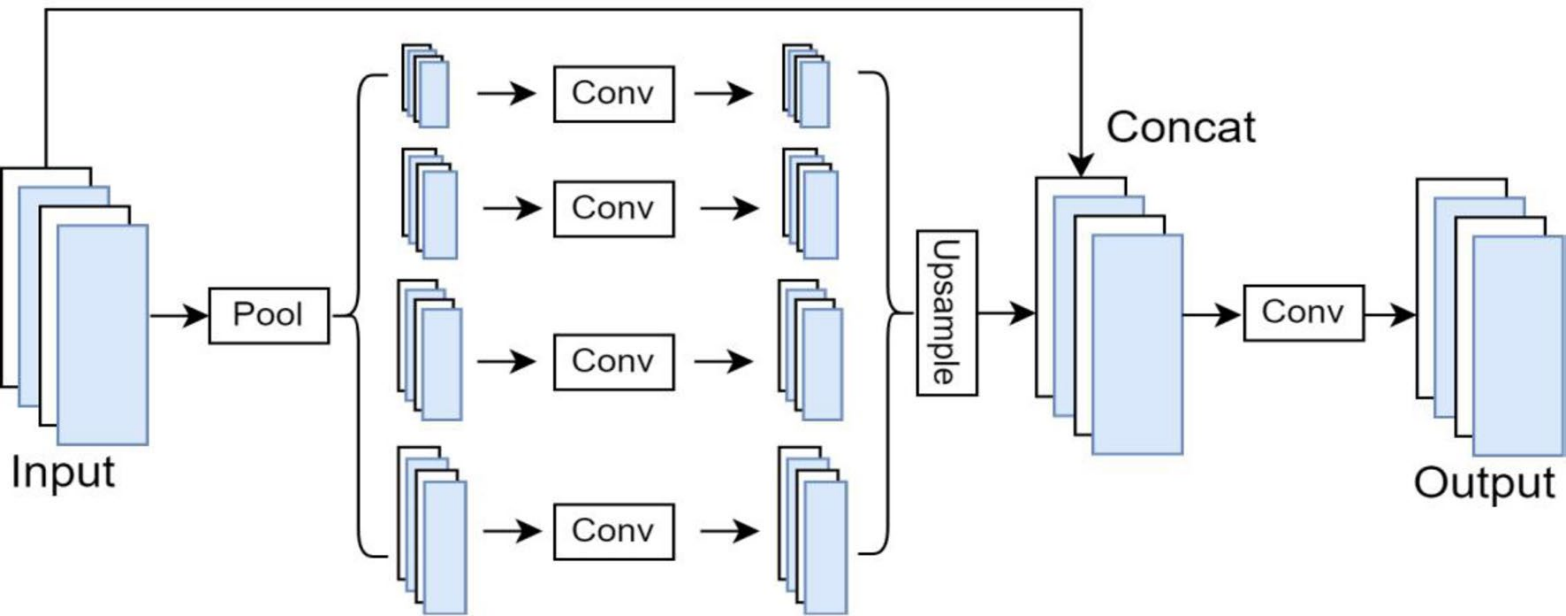
PSPNet Model Architecture^24^.

PSPNet is the Pyramid Pooling Module, which captures multi-scale contextual information from the input image. This module helps the network understand the context and relationships between objects and regions of various sizes within the scene. The PSPNet is shown in Fig. 3.

### U-Net model

**Fig. 4:**
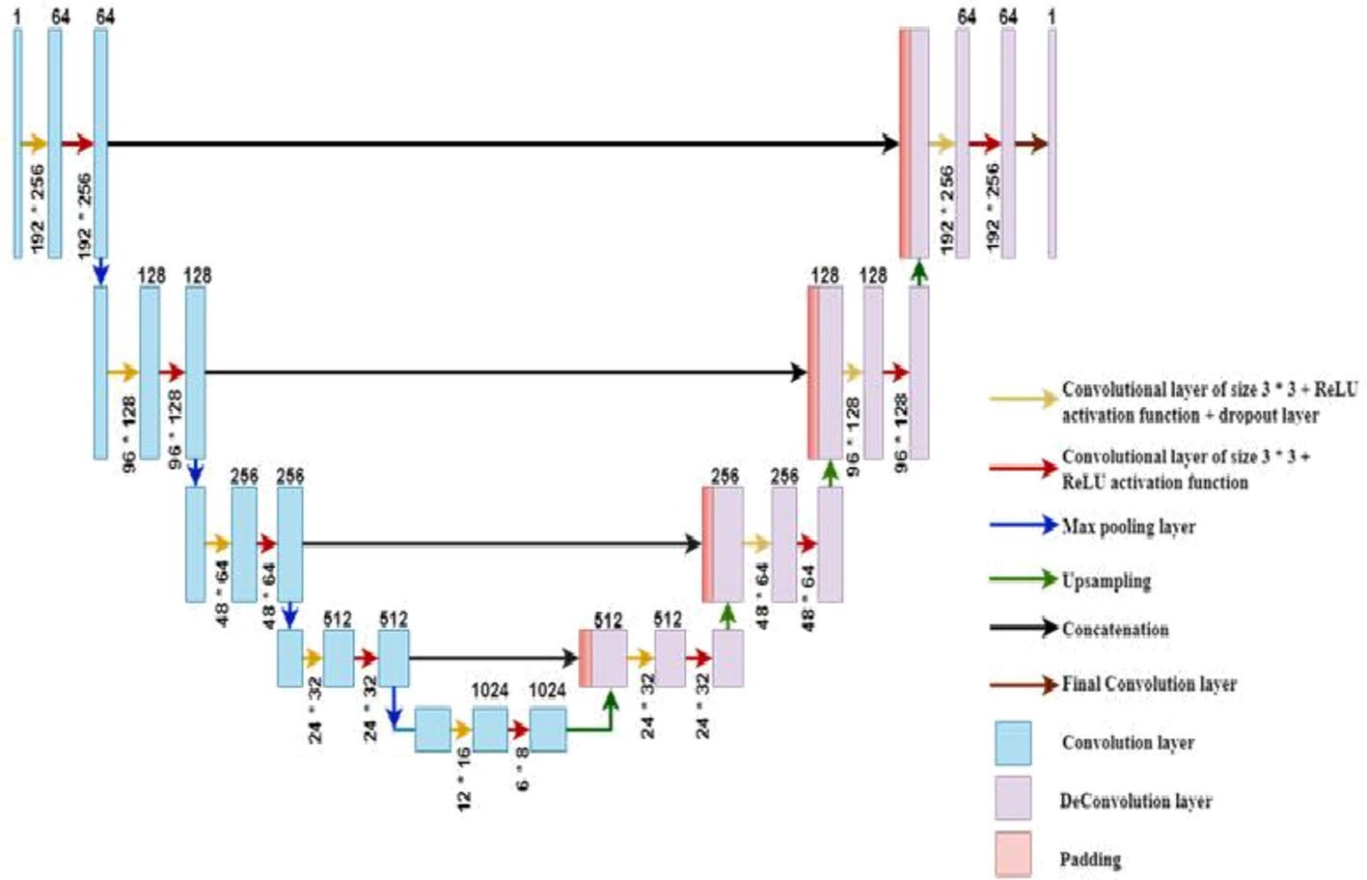
The standard U-Net Model Architecture^25^.

Proposed in^25^, U-Net is a convolutional neural network (CNN) architecture primarily used in biomedical image analysis tasks like the segmentation of cells and tissues from microscopy images. Figure 4 illustrates the U-shaped design of the U-Net architecture, which consists of an encoder and a decoder. U-Net is an encoder-decoder network. In the encoder stage, the image input is processed using convolutional and pooling layers to extract particular features; in contrast, the decoder takes the processed information and reintegrates it back through upsampling stages to create a segmentation map corresponding to the original input image size. U-Net was first conceived for segmentation of medical images, but has been broadened and used in other areas, such as road segmentation in aerial images and cell nucleus segmentation, among others.

### Proposed LinkNet-152 model for spine segmentation

**Fig. 5:**
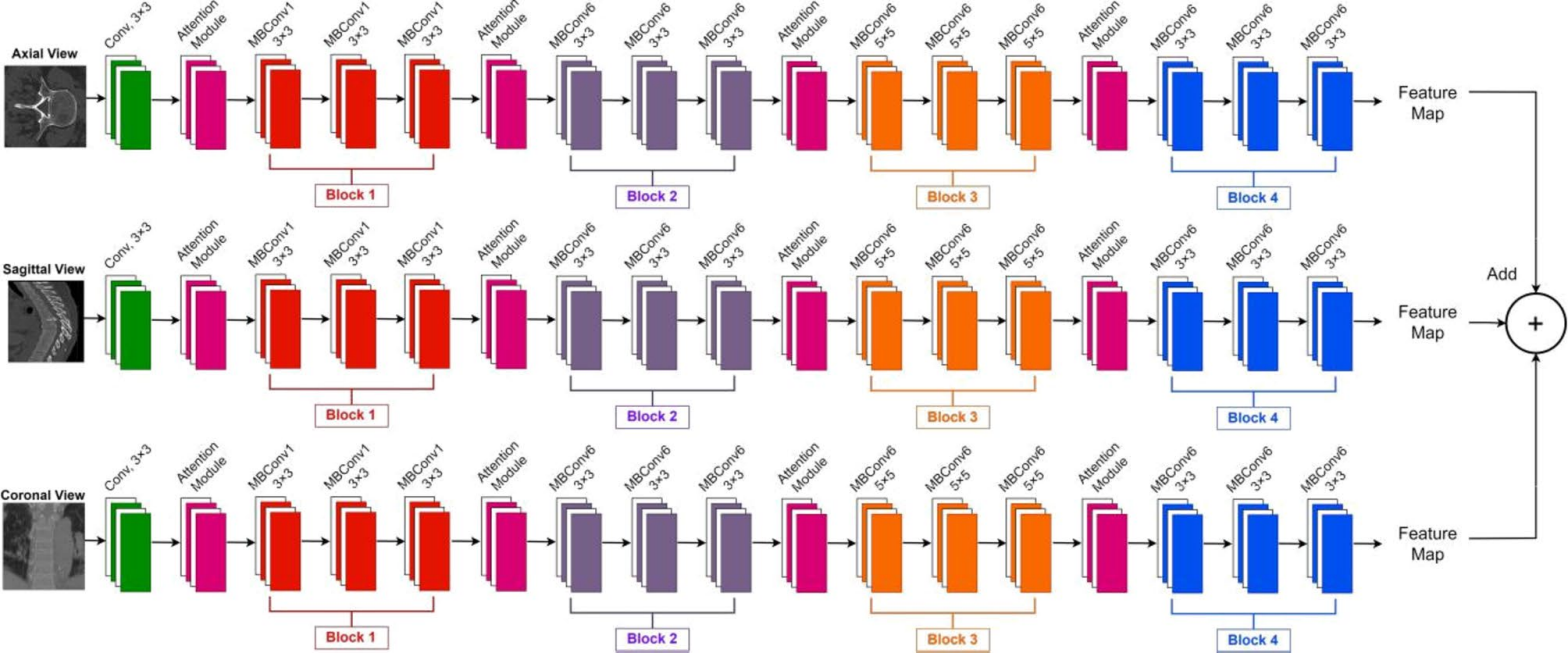
The modified architecture of EfficientNetB7 used in this research.

**Fig. 6:**
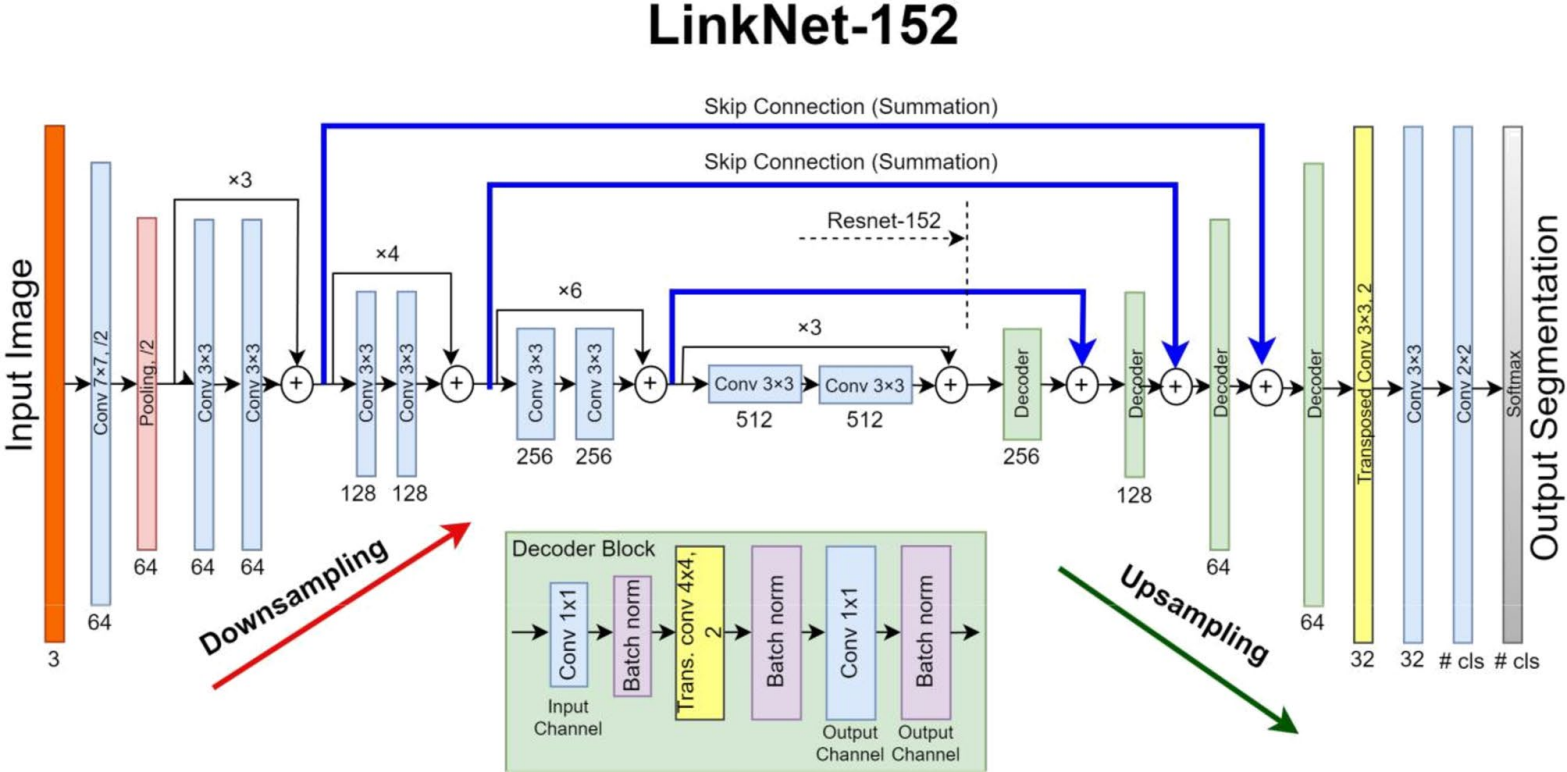
The proposed LinkNet-152 used in this research.

The proposed model is a combination of EfficientNetB7, ResNet152, and LinkNet. The EfficientNetB7 consists of 7 different blocks with multiple convolution operations. However, the proposed approach is an encoder-decoder-based architecture in which each of the first four blocks of EfficientNetB7 is followed by an attention module. The modified version of the EfficientNetB7 is shown in Fig. 5. In the proposed LinkNet-152 model, the first four blocks of EfficientNetB7 were modified by integrating attention modules to enhance feature extraction for spine segmentation. These attention modules focus on the regions of interest by emphasizing critical features and suppressing irrelevant ones. By refining the extracted features, the attention mechanism ensures that the model captures both local and global contextual information, which is crucial for accurately delineating the complex structures of the spine. The 3D CT image consists of axial, sagittal, and coronal views. The features from each view are extracted by using a separate modified EfficientNetB7. These features are concatenated and given.

to the LinkNet model for final prediction. The standard LinkNet model used ResNet-34 for feature extraction. In the proposed approach, the ResNet-34 is replaced with the ResNet-152 which is more powerful in extracting prominent deep features from the dataset. The proposed LinkNet-152 is used to predict the final segmentation of the spine. After that, these features are passed to the proposed LinkNet-152 for spine segmentation. The ResNet152 is used as an encoder for feature extraction instead of ResNet34. ResNet152 is known for its deep structure, which is made possible by the use of residual connections, also known as skip connections or shortcut connections. The ResNet152 is used to extract deep features from the input feature maps. The proposed LinkNet-152 architecture is shown in Fig. 6.

### A gradient sensitivity based network pruning

Semantic segmentation of medical images, particularly spine segmentation, is crucial for accurate diagnosis and treatment planning. HyperDenseNet has proven to be highly effective in this domain. However, the large number of parameters in HyperDenseNet models can lead to substantial computational and memory requirements, making deployment on resource-limited devices challenging. Network pruning, a technique that reduces model complexity by eliminating less significant parameters, offers a promising solution to this problem^26,27^. In this paper, we introduce a pruning method based on gradient sensitivity, specifically designed to enhance the performance of the HyperDenseNet for spine segmentation tasks. This approach identifies and prunes filters that contribute least to the model’s output, thereby optimizing the network for both efficiency and accuracy. The following sections detail the steps involved in this pruning technique, including initial training, gradient sensitivity computation, filter pruning, fine-tuning, and evaluation.

#### Step 1: train initial model

The process begins by initializing a U-Net model, a widely used architecture for semantic segmentation due to its robust encoder-decoder structure. The model is trained on a spine segmentation dataset, where the input images are processed through the network to produce segmentation masks. The training objective is to minimize the binary cross-entropy (BCE) loss function:

1 $$\:\text{L}\left(\text{y},\:\text{y}^{\wedge}\right)=\:-\frac{1}{\text{N}}{\Sigma\:}\text{i}\text{N}=1\text{y}\text{i}\text{l}\text{o}\text{g}\left(\text{y}^{\wedge}\text{i}\right)\:+\:(1\:-\text{y}\text{i})\text{l}\text{o}\text{g}(1\:-\text{y}^{\wedge}\text{i})$$

Here, y and yˆ represent the ground truth and predicted segmentation masks, respectively. Over several epochs, the model adjusts its weights to learn the mapping from input images to accurate segmentation masks. The trained model is then saved for the pruning phase.

#### Step 2: compute gradient sensitivity

After training, the model is evaluated to calculate the gradient sensitivity of each filter in the convolutional layers. This involves setting the model to evaluation mode and performing a forward pass to obtain the segmentation output and.

compute the loss. Subsequently, a backward pass is conducted to determine the gradients of the weights for the loss. The gradient sensitivity of a filter F is calculated as:

2 $$\:S\left(F\right)\:=1w\in\:F\:|\partial\:\:L|\left|F\right|\partial\:\:w$$

where |F| denotes the number of weights in the filter F, and ^*∂L*^ is the gradient of the loss concerning each weight w. This.

sensitivity measure indicates the relative importance of each filter in the network.

#### Step 3: prune filters based on sensitivity

The filters with the lowest sensitivities are identified for pruning using the computed gradient sensitivities. These filters are considered redundant as they contribute the least to the model’s performance. The pruning amount p is a predefined parameter that specifies the proportion of filters to be removed. The filters to be pruned, denoted as P, are those whose sensitivities fall below a certain threshold:

3 $$\:\text{P}\:=\:\left\{\text{F}\text{i}|\text{S}\left(\text{F}\text{i}\right)\hspace{0.17em}\le\:\hspace{0.17em}\text{t}\text{h}\text{r}\text{e}\text{s}\text{h}\text{o}\text{l}\text{d}\right\}$$

The weights and biases of the selected filters are set to zero, effectively eliminating their influence on the network.

#### Step 4: Fine-tune the pruned network

Following pruning, the network is fine-tuned to adjust the remaining weights and compensate for any performance degradation caused by pruning. Fine-tuning involves retraining the pruned model on the same dataset with a reduced learning rate to refine the weights. The loss function during fine-tuning may include a regularization term to prevent overfitting:

4 $$\:{\text{L}}^{{\prime\:}}\left(\text{y},\:\text{y}^{\wedge}\right)\hspace{0.17em}=\hspace{0.17em}\text{L}\left(\text{y},\:\text{y}^{\wedge}\right)+\:\:\text{i}\text{N}=1\text{R}\left(\text{w}\text{i}\right)$$

where λ is a regularization parameter and R(W_i_) is a regularization term. This step ensures that the pruned model regains or even improves its segmentation accuracy.

#### Step 5: evaluate and save the pruned model

The final step is to evaluate the pruned and fine-tuned model on a validation dataset to ensure it retains high performance.

### Experimental setup

#### Dataset definition

This research employed the publicly accessible datasets VerSe 2020 and VerSe 2019^28–30^for model evaluation. The dataset is available at the OSF repository^31^. The VerSe 2020 dataset contains 300 CT images, complete with annotations. The VerSe.

**Table 1:** Dataset description of verse 2020 and verse 2019 dataset.

Dataset	Spine Tract	Total Image	Modality
VerSe 2020	Whole Spine	300	CT
VerSe 2019	Whole Spine	160	CT

2019 dataset includes 160 CT images along with centroids and segmented masks. Detailed information about both the VerSe 2020 and VerSe 2019 datasets is provided in Table 1.

### Evaluation metrics

#### Dice coefficient score

The Dice coefficient is used to quantify how well the predicted (segmented) region aligns with the ground truth (the actual segmentation). It provides a single numerical value that reflects the degree of agreement or overlap between these two.

5 $$\:\text{D}\text{S}\text{C}\:=\:2\left(\text{P}1\:\times\:\text{P}2\right)(\text{P}1\hspace{0.17em}+\hspace{0.17em}\text{P}2)$$

where P1 is the actual ground truth and P2 is predicted ground truth.

#### Intersection over union

IoU is commonly used to assess the performance of algorithms and models in tasks where precise localization or region-based accuracy is essential. It is particularly useful in object detection, instance segmentation, and semantic segmentation tasks.

6 $$\cup\:\text{I}\text{o}\text{U}\:=\text{A}\:\cap\:\text{B}\text{A}\:\cup\:\text{B}$$

where A is the actual ground truth and B is predicted ground truth.

### Pre-processing

In order to enhance the quality of the dataset, certain techniques referred to as pre-processing are exercised. In order to feed images into the deep learning model, all images are resized to 256 × 256. The dataset is split into two parts in which 80% of the dataset is used for training while the remaining 20% dataset is for testing for the proposed model. The model training is done in 100 epochs with an initial learning rate of 0.001. The learning rate was decreased by a multiplication factor of 0.1 after every 10 epochs where there was no enhancement in the course of training. The processing of 3D CT images is challenging owing.

to several factors including higher possibilities of complexity. There are very high amounts of data that require storage and processing power making it ill-advised to perform such tasks. Table 2 shows the hyperparameters of the proposed approach.

**Table 2:** Hyperparameters for deep learning model for spine segmentation.

Hyperparameter	Typical Range / Value
Learning Rate	0.001
Batch Size	8
Epochs	100
Optimizer	Adam
Dropout Rate	0.3
Loss Function	Dice Loss
Activation Function	ReLU
Input Image Size	256 × 256
Early Stopping Patience	10

### Data augmentation

To increase the performance of the machine learning models, data augmentation methods are widely used^32^. The dataset is pre-processed by using smoothing, clamping, and reorienting. In data augmentation, smoothing, clamping, and reorienting are techniques used to enhance the robustness and diversity of training data. Smoothing involves applying filters to reduce noise and variability in images, helping the model generalize better by focusing on essential features. Clamping adjusts pixel values to stay within a specified range, preventing extreme values that could lead to overfitting or artifacts in the data. Reorienting rotates or flips images to simulate different perspectives and orientations, increasing the model’s ability to recognize patterns and objects from various angles. Together, these methods improve the model’s performance by providing a richer and more varied dataset.

#### Impact of data augmentation on model robustness

Data augmentation techniques were an integral part of enhancing the performance of the deep learning model for spine segmentation. These techniques aimed to artificially increase the diversity of the training dataset, enabling the model to generalize better to unseen data. Specifically, three main augmentation strategies were employed: smoothing, clamping, and reorienting.

Smoothing involved applying filters to reduce noise and variability in the images, allowing the model to focus on essential features and ignore irrelevant artifacts. Clamping adjusted pixel values to remain within a specified range, which helped mitigate the impact of extreme values that could introduce artifacts or lead to overfitting. Reorienting simulated different perspectives and orientations by rotating or flipping images, ensuring the model could recognize patterns and structures regardless of their spatial orientation.

The application of these techniques significantly improved the model’s robustness. First, it enhanced the model’s general-ization ability, making it more adept at handling variations in CT images, such as differences in orientation, noise levels, and imaging quality. Second, the model achieved higher segmentation accuracy, as evidenced by an increase in key performance metrics like the Dice coefficient and Intersection over Union (IoU). Third, the augmentation strategies reduced overfitting by exposing the model to a wide variety of data variations, which was reflected in the reduction of validation loss during training.

The techniques improved the model’s robustness against noise and imaging artifacts. Smoothing and clamping ensured the model could effectively segment spine structures even in cases of suboptimal scan quality. Reorienting further contributed to this robustness by enabling the model to recognize anatomical features from various angles. Notably, the augmented dataset also enhanced the model’s ability to detect intricate boundaries, which is crucial for segmenting irregular or overlapping vertebrae, as well as structures with fractures or atypical shapes.

## Results and discussion

This part highlights the outcome of the proposed models for spine segmentation. The performance of the models is assessed on VerSe 2020 and VerSe 2019 datasets. The detailed information regarding the experiments performed in the study is given here in complete detail. The experiments were carried out under the supervision of a medical doctor who has practiced medicine for three years after graduating with an MBBS degree from Shandong First Medical University in China and was on clinical attachment at Sahiwal Medical College (SLMC). All methods were carried out by appropriate guidelines and regulations. The experimental protocol received approval from the supervising medical doctor.

**Table 3:** Comparison of the deep learning models for semantic segmentation.

Metrics	Link-Net	FPN-Net	PSP-Net	U-Net
DSC (%)	94	92	92.5	93
Jaccard (%)	90	89	90	90
Training Loss (%)	0.003	0.003	0.003	0.003
Validation Loss (%)	0.004	0.007	0.006	0.005

### Results of semantic segmentation models

This section compares four models based on loss analysis, the Jaccard index, and the Dice coefficient. Table 3 presents the findings for these metrics across the models. The Dice coefficient scores for LinkNet-34, FPN-Net, PSP-Net, and U-Net are 94%, 92%, 92.5%, and 93%, respectively. The Jaccard index results show that LinkNet-34, PSP-Net, and U-Net all achieve a value of 0.9. Consequently, the Dice coefficient is used to evaluate model performance, with LinkNet-34 emerging as the best performer in this regard. Additionally, the validation losses for the models are as follows: LinkNet-34 (0.004%), FPN-Net (0.007%), PSP-Net (0.006%), and U-Net (0.005%). From Table 3 it can be concluded that the LinkNet-34 model gives the highest value of the dice coefficient of 94%. Although the value of the Jaccard index is 90% on LinkNet-34, PSPNet, and U-Net models, the value of the loss is approximately the same on all four models.

#### Results of all encoder models

In machine learning, encoders play a crucial role in various tasks, and their impact depends on the specific context in which they are used. Encoders are a fundamental component of neural networks and other machine learning models, and they are responsible for transforming raw data into a format that is suitable for learning and analysis. The results of all the encoder models are given in Table 4.

ResNet34^33^ is a widely used deep convolutional neural network (CNN) architecture for image classification, known for its 34-layer depth. It employs a technique called residual connections to mitigate the vanishing gradient issue. These residual links bypass certain layers of the network, allowing for more efficient training and improving gradient flow. ResNet34 is known for its fast training process and high prediction accuracy, making it an effective architecture. The model achieves a Dice Similarity Coefficient (DSC) of 91%, a Jaccard index of 88%, with training and validation losses of 0.004% and 0.007%, respectively.

MobileNetV2^34^ is a popular CNN-based architecture, particularly favored for mobile devices due to its compact size (with only 53 layers) and impressive performance. It leverages a technique called inverted residuals to optimize efficiency. This type of residual connection utilizes a narrow bottleneck layer, which reduces the number of parameters and operations, all while maintaining accuracy. The architecture achieves strong performance metrics, with a Dice Similarity Coefficient (DSC) of 92%, a Jaccard index of 89%, a training loss of 0.003%, and a validation loss of 0.006%.

Residual connections are a key feature of ResNet50^35^to avoid the vanishing gradient problem in deep CNNs. They enhance the definition of gradients while propagating it through the network while training it. ResNet50 also reduces the parameter size by using bottleneck layers, which have fewer channels than the previous layer. Quantitative assessment metrics include 90% Dice Similarity Co-efficient (DSC), 88% Jaccard index, and training as well as validation losses of 0.005% and 0.006% respectively. The outcomes achieved by the EfficientNetB7 model^36^ are represented as a Dice Similarity Coefficient with Jaccard indices, training loss, and validation loss of 93%, 90%, 0.002%, and 0.006% respectively.

Different encoders: ResNet34, MobileNetV2, ResNet50, and EfficientNetB7 are applied for spine segmentation in CT images.

over the same base architecture LinkNet-34. The encoders are assessed using Different metrics: the Dice Coefficient, the Jaccard Index, and loss as provided in disjoint. Table 4. EfficientNetB7 stands out as the top rated and most efficient encoder available that facilitates the downgrading process in the LinkNet-34 semantic segmentation model for better comprehension and appreciation of spatial arrangement and higher-level features, thus performing precise pixel-wise semantic segmentation.

### Results of multiple optimizers

In the last part, it was concluded that among all the semantic segmentation models, LinkNet-34 is the best with EfficientNetB7 as the encoder which produces the model with the highest dice coefficient and jaccard index value. An optimizer is one of the crucial components of semantic segmentation as it updates the parameters of the model during the training process. Optimizers are the algorithms that determine what is the weight and biases change of the model to minimize the loss function which is the difference between the actual segmentation and the predicted segmentation. The choice of the optimizer can change the process of training and also the output of the final segmentation. In this section, however, the LinkNet-34 semantic segmentation model was tested on three optimizers named Adam, RMSProp, and Adamax in order to achieve the most suitable optimization for the given semantic segmentation task. The results from all the optimizers are given in Table 5 and explained below.

**Table 4:** Comparison of the deep learning encoder models for semantic segmentation.

Metrics	ResNet34	MobileNetv2	ResNet50	EfficientNetB7
DSC	91	92	90	93
(%)				
Jaccard	88	89	88	90
(%)				
Training	0.004	0.003	0.005	0.002
Loss				
(%)				
Validation	0.007	0.006	0.006	0.006
Loss				
(%)				

**Table 5:** Comparison of the deep learning models for semantic segmentation.

Metrics	Adam	RMS-prop	Adamax
DSC (%)	91	92	91
Jaccard (%)	90	89	89
Training Loss (%)	0.002	0.002	0.002
Validation Loss (%)	0.006	0.006	0.007

### RMSprop optimizer analysis

Within this component, the highest-yielding LinkNet-34 architecture with EfficientNetB7 as an encoder is analyzed with respect to the performance of an optimizer called RMSProp. In the following Table 5, the metrics results, including loss and Jaccard index, as well as the Dice Coefficient with respect to the use of RMSProp over the course of 25 epochs are presented. 5. In this scenario, the training and validation losses are 0.002 and 0.006, respectively. The assessment of the Jaccard index and dice coefficient over 25 periods. The value of the graph remains unchanged beyond the seventh epoch, where the Jaccard index and Dice coefficient attain the peak values of 89% and 92%, respectively.

#### Adamax optimizer analysis

This part elaborates on the simulation enhancement of the best LinkNet-34 model with EfficientNetB7 encoder and Adamax optmizer. Table 5 presents the loss, Jaccard index, and Dice Coefficient values for the Adamax optimizer over 25 epochs. The recorded validation loss is 0.007 while the training loss is 0.002. The performance metrics of the Jaccard index and Dice Coefficient, upon training for 25 epochs, was 89% and 91% respectively.

#### Adam optimizer analysis

In this section, the best performing LinkNet-34 model with EfficientNetB7 as the encoder is simulated with Adam optimizer.

Table 5 gives the loss, Jaccard index, and dice coefficient values of the Adam optimizer for 25 epochs.

In order to determine which of the optimizers performed best — RMSProp, Adamax, or Adam — a study of the results obtained was carried out. The Jaccard index achieved for the three optimizers was 89%, 89%, and 90% respectively, and Correspondence Coefficient values were 92%, 91%, and 91% respectively. The comparative analysis shows that the best results were obtained when employing the Adamax optimizer which gave Jaccard and Dice Coefficient scores of 89% and 91%. Therefore, it can be stated that the use of LinkNet-34 with EfficientNetB7 encoder and Adamax optimizer gives the best result in terms of distinguishing predicted segmentation from the ground truth mask in semantic segmentation.

## Results with preprocessing and data augmentation

Preprocessing techniques were applied to the dataset to enhance the quality of the input images and improve model performance. All CT images were resized to a consistent dimension of 256 × 256, ensuring uniform input to the model. The noise reduction techniques such as smoothing were employed to minimize artifacts and variability in the images. The dataset was normalized to maintain pixel values within a standard range, improving numerical stability during training. The preprocessing pipeline also included intensity normalization to enhance contrast and preserve essential details in the spine structures. These preprocessing steps contributed to better segmentation accuracy by improving the clarity of anatomical features, reducing training loss, and enhancing overall robustness. The results showed a noticeable improvement in the Dice coefficient and Intersection over Union (IoU) metrics compared to models trained without preprocessing. The results using preprocessing and data augmentation are given in Table 6.

**Table 6:** Comparison of results with preprocessing and data augmentation.

Methodology	Dice Coefficient (DSC) (%)	Jaccard Index (IoU) (%)	Training Loss (%)	Validation Loss (%)
Preprocessing	93.5	91.2	0.004	0.005
Data Augmentation	94.7	92.5	0.003	0.004

**Fig. 7:**
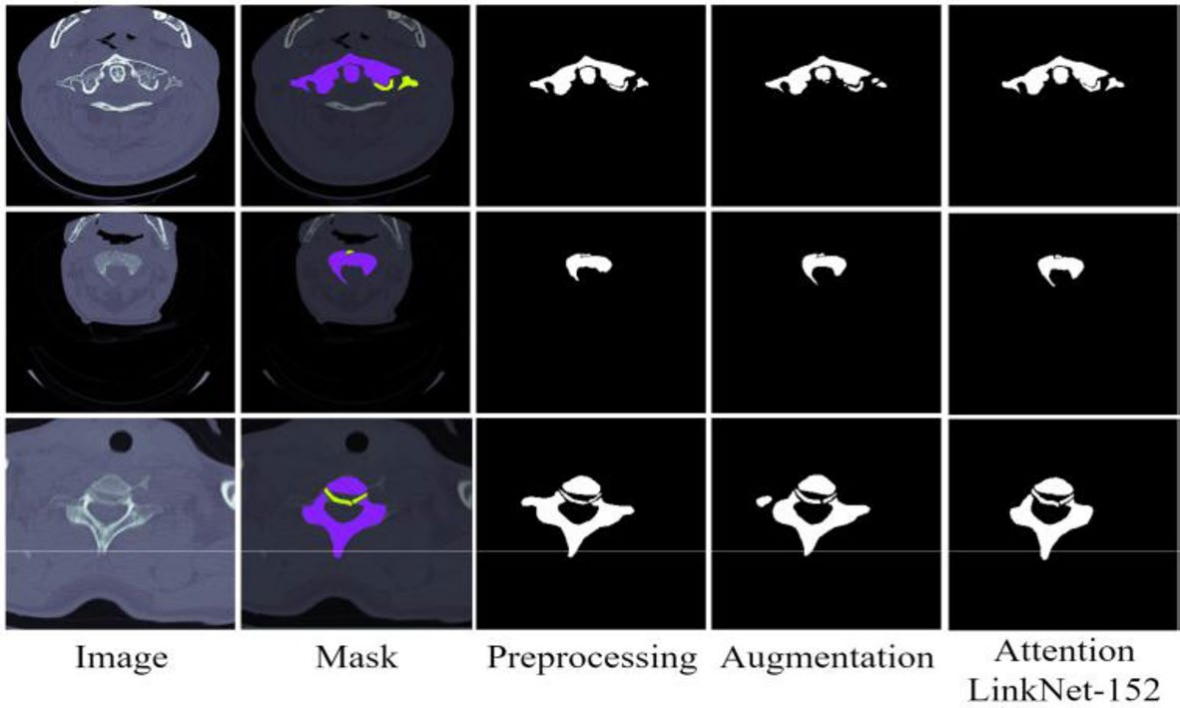
The results of the proposed Attention LinkNet-152 model with preprocessing and data augmentation.

### Visualizing segmentation results

In the last parts of the article, images of the spine obtained from CT scan are segmented using four different semantic segmentation architectures namely LinkNet-34, FPN, PSPNet, and U-Net. From all the models used in the task, LinkNet-34 achieved the best result. After that, LinkNet-34 model performance analysis was done by using four other resource encoders also known as ResNet34, MobileNetv2, ResNet50, and EfficientNetB7 to produce feature maps containing of semantic high-level information as well as spatial information context. It was established that based on the evaluation of the encoders using the Dice Coefficient and the Jaccard Index, EfficientNetB7 was the best encoder. The combination of the LinkNet-34 model with EfficientNetB7 encoders was thereafter subjected to three optimizers: RMSProp, Adamax, and Adam, and it was observed that Adamax produced better performance. Figure 7 shows the visualization of the results of the proposed LinkNet-34 model with the combination of EfficientNetB7 encoder and Adamax optimizer.

### Comparison of the proposed model with other deep learning models

The state-of-art Table 7 presents various studies related to spine segmentation using different techniques and datasets. Most studies involving semantic segmentation typically emphasize performance metrics useful for evaluation purposes such as the Dice Coefficient and Jaccard Index among others. The method put forward for spine segmentation based on LinkNet-152 architecture with an EfficientNetB7 encoder has given excellent performance results with a Dice Coefficient of 96.85% and a Jaccard Index of 95.37%. In^37^ a novel 3D-MRU-Net was used for spine segmentation. The proposed model was encoder-decoder architecture in which residual blocks were used as the encoder and 3D U-Net was used as a decoder. The experiments.

**Table 7:** The comparison of the proposed model with other deep learning models.

Reference	Method	Test Samples	DSC %
37	3D MRU-Net	60	95.19
4	Patch Based DNN	40	90.2
8	U-Net	14	90.4
38	3D V-Net	50	89.7
39	PaDBN	3	92.6
12	U-Net Based CNN	32	86
40	Differentiable Appearance	-	90
2	CHASPPRAU-Net	60	94.58
Proposed	LinkNet-152	80	96.85

were performed on the VerSe’20 and VerSe19 datasets. The results show that 3D-MRU-Net achieves a dice score of 95.19%. Deep learning is an emerging technique for medical analysis. A patch-based deep Learning approach was designed for the CT vertebral segmentation^4^. The approach was tested on the three publicly available datasets VerSe, CSI-Seg, and Lumbar CT. The model performs well as compared to state-of-the-art methods and gives 90.2% accuracy. 60 to 80% adults experience back pain at a certain point in their lives.

In^8^an automated U-Net architecture was presented for the accurate and practical spine segmentation. A customized data set consisting of 344 CT scans was used for experiments. The proposed model achieves 90.4% accuracy. Two systems were developed for the automated segmentation of vertebrae in^38^. In the first method, MLPNN was introduced to classify the vertebra and secondly, APCNN was used for the segmentation of spine Vertebra. A result of 94.2% was achieved by the proposed system. A patch-based deep belief network (PaBDN) was proposed^39^ for automatic segmentation of the spine. The PaBDN automatically selected a feature from the region of interest and an unsupervised algorithm was applied for weight initialization. The proposed model gives a promising result of 86.1% as compared to previous models. Kim et al.^40^ proposed a differentiable appearence method for automated spine segmentation with less dataset. The model learns the image from the input dataset and does not depends on the extracted features. The proposed model was tested on VerSe 2020 dataset which achieved 90% dice score. Saeed et al.^2^ proposed a CHASPPRAU-Net for automated spine segmentation. The proposed model used a modified version of the U-Net as an encoder for feature extraction. These features are passed to the 3D U-Net for final segmentation. The proposed model was tested on VerSe dataset and achieved 94.58% dice score.

## Conclusion

Due to the extraordinary shape and ambiguous boundaries of spines, segmentation is a difficult task. With the possibility that deep learning techniques would be helpful to spine segmentation as it is less prone to any disturbances. This research presents a deep learning encoder-based approach to LinkNet-152 model architecture for the semantic segmentation of spines in CT images. LinkNet-34 is compared with three other models: FPN, U-Net, and PSPNet. The performance of EfficientNetB7 when used as an encoder for LinkNet-152 is also compared with ResNet34, MobileNetv2, and ResNet50. The optimization of the model was evaluated with three optimizers as follows: RMSProp, Adamax, and Adam, with Adamax giving the best results of 95.37% mean Jaccard index and 96.85% Dice coefficient.

## Data Availability

This research employed the publicly accessible datasets VerSe 2020 and VerSe 2019 and can be downloaded from this url https://osf.io/nqjyw/.
